# Mechanical needle guidance for ultrasound-guided parasagittal oblique in-plane paravertebral blocks: a cadaveric study

**DOI:** 10.1016/j.bjane.2025.844716

**Published:** 2025-11-28

**Authors:** Hesham Elsharkawy, Ece Yamak Altinpulluk, Tetsuya Shimada, Ilker Ince, Guangmei Mao, Nicolas Mario Mas D Alessandro, Loran Mounir Soliman, Nair Harsha, Marta Kelava, Richard Drake, Xuan Pu, Daniel I. Sessler, Alparslan Turan

**Affiliations:** a Case Western University, Pain and Healing Center, MetroHealth, The MetroHealth System, Case Western Reserve University School of Medicine, Cleveland, OH, USA; b Department of Outcomes Research, Houston, Texas, USA; c Madrid Morphological Research Center, Madrid, Spain; d Ataturk University, Anesthesiology Clinical Research Office, Erzurum, Turkey; e National Hospital Organization, Murayama Medical Center, Department of Anesthesiology, Musashimurayama, Tokyo, Japan; f National Defense Medical College, Department of Anesthesiology, Tokorozawa, Saitama, Japan; g Penn State University Milton S Hershey Medical Center, Department of Anesthesiology and Perioperative Medicine, Hershey, PA, USA; h Cleveland Clinic, Quantitative Health Sciences, Cleveland, OH, USA; i The MetroHealth System, Case Western Reserve University School of Medicine, Department of Anesthesiology and Perioperative Services, Cleveland, OH, USA; j Cleveland Clinic, Department of Cardiothoracic Anesthesiology, Cleveland, OH, USA; k Cleveland Clinic Lerner College of Medicine of Case Western Reserve University, Cleveland Clinic, Cleveland, OH, USA; l Anesthesiology Institute, Cleveland Clinic, Department of General Anesthesiology, Cleveland, OH, USA

**Keywords:** Anesthesia, Cadaver, Thoracic Surgery, Ultrasonography

## Abstract

**Background:**

Paravertebral blocks provide analgesia for a range of thoracoabdominal surgeries. However, visualizing the needle tip during the procedure can be challenging, especially for clinicians with limited experience, because the target is deep. We therefore tested the primary hypothesis that needle guidance by the Infiniti Plus system improves ultrasound visualization of the needle tip during thoracic paravertebral blocks performed by novice residents.

**Methods:**

Nineteen clinical anesthesia residents each performed 20 bilateral ultrasound-guided thoracic paravertebral blocks (T2–T11) on 17 unembalmed cadavers, with and without the use of a fixed-angle mechanical needle guide in a randomized crossover design. The primary outcome, percent perfect needle visibility, was compared between guided and unguided methods using a paired *t*-test. Secondary outcomes, including time to needle visualization, number of needle insertion attempts, and subjective ease-of-use ratings, were analyzed using paired *t*-tests and Wilcoxon signed-rank tests, respectively. Inter-rater reliability for overall perception ratings was assessed using the Intraclass Correlation Coefficient (ICC).

**Results:**

There were no significant differences in needle-target visualization (62% ± 17% with guidance vs. 64% ± 18% without, p = 0.15), time to target (HR = 1.00 [95% CI 0.86–1.16], p = 0.99), procedural difficulty scores, or number of insertion attempts between guided and unguided blocks.

**Conclusion:**

The Infiniti Plus mechanical needle guide did not demonstrate improved ultrasound needle tip visualization during thoracic paravertebral blocks performed by novice clinicians in cadavers.

## Introduction

Paravertebral blocks are an advanced regional anesthesia technique entailing unilateral block of spinal nerve roots as they exit intervertebral foramina.^1^, ^2^, ^3^, ^4^ Paravertebral blocks provide good analgesia for thoracic, breast,^5^^,^^6^ abdominal,^7^ and renal surgeries in adults and children.^8^^,^^9^

Ultrasound visualizes the anatomy of the paravertebral space and the real-time distribution of local anesthetics.^10^^,^^11^ As expected, ultrasound guidance improves block success^12^ and reduces complications.^13^ However, ultrasound-guided paravertebral block requires excellent hand-eye three-dimensional coordination extrapolated from a two-dimensional ultrasound image. Even for experienced practitioners, the greatest challenge lies in visualizing the needle tip while advancing it towards the target.^11^ Advancing the needle without needle tip visualization may lead to vascular, neural, or visceral injury.^13^, ^14^, ^15^ Poor image quality in paravertebral blocks leads to higher failure rates.

**Figure 1:**
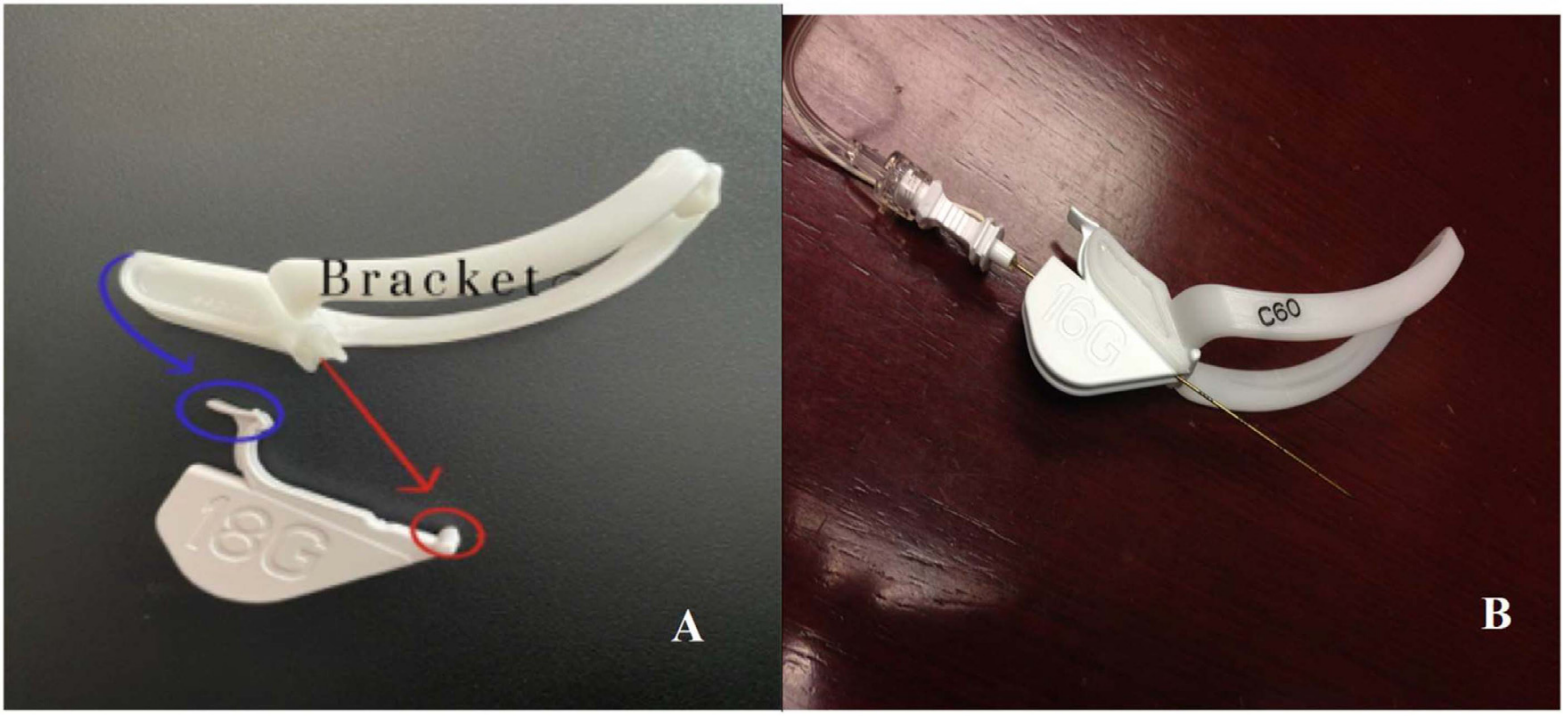
(A) Infiniti Plus needle guidance system; blue circular segment fits in the top of the bracket; the red circular segment part fits in the bottom of the bracket. (B) Infiniti Plus needle guidance system with the needle fits in the bracket.

Needle guidance techniques have been developed to improve the accuracy and safety of ultrasound-guided procedures by providing real-time visualization of the needle trajectory. Infiniti Plus (In-plane, CIVCO Medical Solutions, Coralville, Iowa) is a mechanical guidance system designed to improve ultrasound visualization (Fig. 1A‒B). However, this system has not been tested for the paravertebral blocks by novice clinicians.

We thus evaluated whether mechanical needle guidance improves ultrasound visualization, procedural performance, and efficiency when used by novice anesthesia residents in a cadaver paravertebral block model. Specifically, we tested the primary hypothesis that mechanical needle guidance by Infiniti Plus (In-plane, CIVCO Medical Solutions, Coralville, Iowa) improves ultrasound visualization of the needle tip by novice clinicians. Secondarily, we tested the hypotheses that mechanical needle guidance reduces the time required to reach the target. Exploratory outcomes were procedural difficulty and the required number of needle insertion attempts.

## Materials and methods

The Cleveland Clinic Institutional Review Board approved the participation of residents and use of fresh cadavers (n° 17-013, date 2016). All cadavers were legally donated to the Department of Anatomy at the Cleveland Clinic Lerner College of Medicine at Case Western Reserve University. There is no clinical trial registration since this study was a study in cadavers.

Cleveland Clinic Anesthesiology Institute Clinical Anesthesia year-1 (CA-1) and year-2 (CA-2) residents were recruited via e-mail. Written informed consent was obtained from all the residents who wanted to participate in the study. Results were coded to protect residents who performed poorly. Residents were *a priori* provided with educational materials about ultrasound-guided paravertebral blocks and were thereafter required to score at least 7 out of 10 questions correctly on an examination of anatomy and ultrasound imaging pertinent to paravertebral blocks.^16^

Bodies were donated to the Department of Anatomy at Cleveland Clinic for scientific research and educational purposes with relevant consent. We used a total of 17 unembalmed cadavers, male and female, and with a range of body habitus. We excluded cadavers with known thoracic spinal deformities, thoracic cavity disease, and those who had previous thoracic spinal surgery. One cadaver was used for each participating resident, each on a separate day. Cadavers were maintained at ambient temperature for at least 12 hours before being studied.

### Protocol

**Figure 2:**
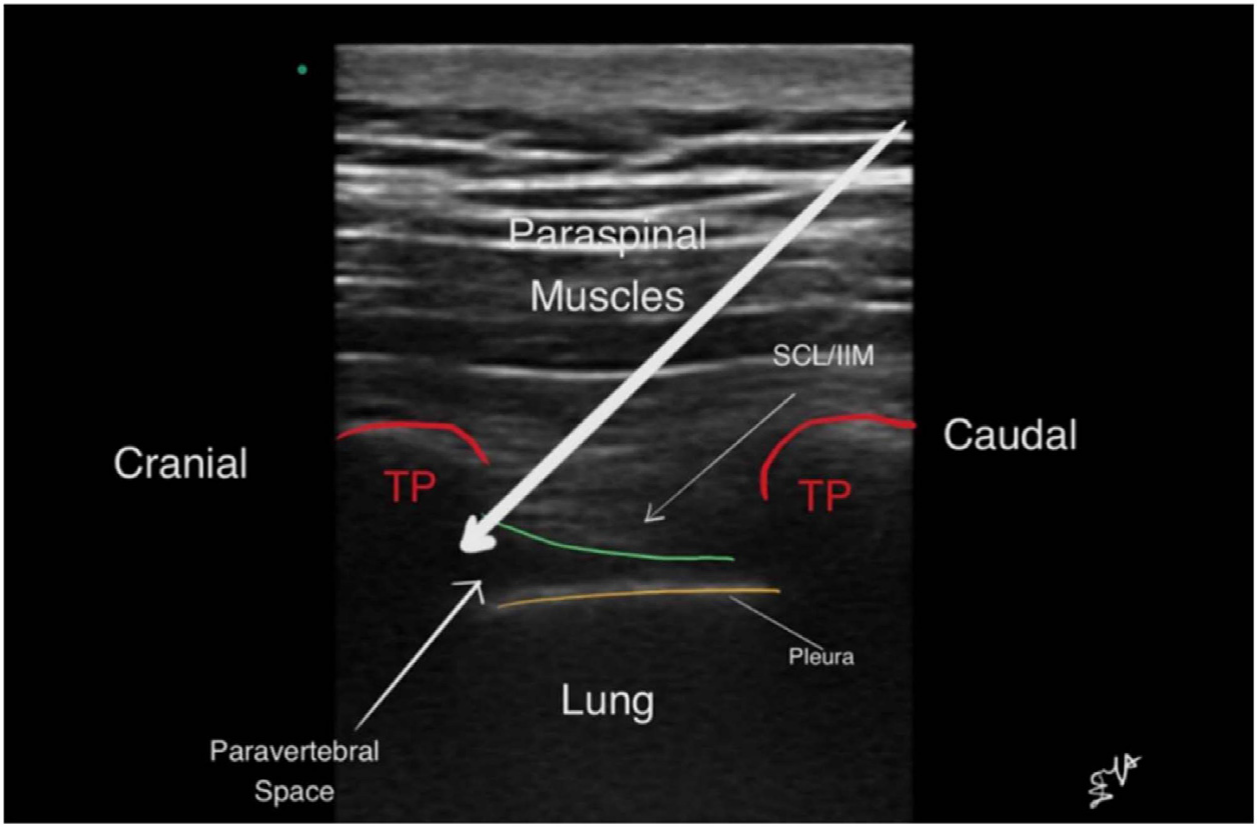
Ultrasound-guided paravertebral block using paramedian oblique sagittal scan without needle guidance at T4. The long white arrow represents the needle inserted parasagittal in-plane technique. TP, Transverse Process; SCL, Superior Costotransverse Ligament; IIM, Internal Intercostal Membrane.

The study was conducted in the anatomy laboratory at the Cleveland Clinic Main Campus. The cadavers were positioned prone with blankets under the abdomen to flex the thoracolumbar spine. The relevant skin landmarks, including thoracic spinous processes, were identified and marked. A point lateral to the tip of the spinous processes, a curvilinear ultrasound transducer (2‒5 MHz) (M-Turbo USG system; Sonosite, Bothell, WA, USA) was used. The transducer was positioned parallel to the spinous process. The transverse process, the costo-transverse ligament and the parietal pleura were identified. Afterward, the probe tilted obliquely in the lateral direction to improve visualization of the parietal pleura. A 125 mm, 18-gauge echogenic Touhy needle (Pajunk Tuohy Sono, Geisingen, Germany) was used for all blocks. Needles were inserted at the caudal end of the ultrasound transducer. Using an in-plane technique, needles were directed towards the costo-transverse ligament. Passage of the needle through the costo-transverse ligament was associated with a tactile loss of resistance. When the resident believed the needle was properly positioned, 2‒5 mL of normal saline was injected into the space. Appropriate spread was identified as anterior displacement of the parietal pleura. Ultrasound imaging and videos of the paravertebral block were recorded for later evaluations (Fig. 2).

In this randomized, paired design study, 19 novice anesthesia residents performed bilateral paravertebral blocks (T2–T11) on cadaver specimens, totaling 20 blocks per resident. Each side of the cadaver was randomly assigned to either the mechanical needle guidance system (Infiniti Plus in-plane, CIVCO Medical Solutions, Coralville, Iowa) or the free-hand technique, with the contralateral side serving as the paired control. Within-subject pairing effectively controlled for inter-individual variability, ensuring that observed differences in procedural outcomes were attributable to the guidance method rather than to anatomical or personal factors. Allocation was based on computer-generated randomization that was presented to the participating resident in a sealed envelope method on the day of the procedure. Allocation was thus concealed as long as it was practical.

### Measurements

Demographic and morphometric characteristics of the cadavers were recorded. Relevant information about participating residents was similarly recorded.

Our primary outcome was the percentage of perfect visualization, defined as the percent of the time with perfect needle tip visibility. Four experienced assessors from our Regional Anesthesia group with experience in ultrasound-guided paravertebral blocks in at least 40 patients were asked to independently evaluate the percentage of the time that needle visualization was perfect. Assessors also gave their overall perception of needle visibility, which was evaluated on a five-point Likert scale. Assessors were blinded to the needle guidance system used for each paravertebral nerve block, but residents doing the injections were not.

Our secondary outcome, time to target, was defined as the time from the needle skin puncture to target site injection of saline in seconds as recorded by research assistants. Exploratory outcomes included the assessors’ overall perception of needle visibility, the number of skin needle attempts, and procedural difficulty. The number of skin puncture attempts was defined as pulling block needle back to skin and reinserting it in the same or a different location. Overall procedural difficulty was rated by residents for needle-guided method and unguided method separately on each subject they performed blocks on after completing the blocks. Therefore, there were 2 procedural difficulty scores in total on each cadaver: one for the guided method and the other for the unguided method. The scale of procedural difficulty ranged from 1 (easy) to 10 (extremely difficult).

### Statistical analysis

Prior to analysis, we assessed the normality of residuals and variables involved in the Generalized Estimating Equations (GEE) and Intraclass Correlation Coefficient (ICC) calculations using QQ-plots. No major deviations from normality were observed, supporting the assumptions of the applied statistical methods.

Exploratory analyses, including comparisons of procedural difficulty and the number of needle insertion attempts, were conducted descriptively. No p-values were calculated for these comparisons, as they were not pre-specified in the statistical analysis plan. Instead, we report descriptive statistics and 95% Confidence Intervals to provide an overview of these outcomes.

### Primary analysis

The primary outcome was the percentage of perfect needle visualization, defined as continuous, real-time identification of the entire needle shaft and tip throughout advancement. The needle appeared as a hyperechoic line within the ultrasound beam plane, without dropout or ambiguity. This definition aligns with established criteria for optimal needle visualization in ultrasound-guided procedures.

For each thoracic level (T2–T11) and side (left/right) in each cadaver, assessments from four independent observers were averaged to determine the final percentage of perfect visualization.

A generalized linear model with an identity link was fitted using the Generalized Estimating Equation (GEE) method, assuming an exchangeable covariance structure within subjects. The model included covariates for thoracic level, block side, and resident training level (CA-1 vs. CA-2) to adjust for potential confounding factors.

As a sensitivity analysis, comparisons were repeated using each assessor’s evaluations separately. The distributions of the averaged and individual assessments were approximately normal, as evidenced by Q-Q plots.

“Target arrival” was operationally defined as the point at which the needle tip was visualized in-plane at the intended paravertebral target site under real-time ultrasound guidance, immediately followed by the observation of pleural displacement upon saline injection. This dynamic assessment ensured accurate needle placement and was consistently applied across all procedures.

### Assessing inter-rater reliability

We assessed Inter-Rater Reliability (IRR) for the primary outcome-percent perfect visualization-using two forms of the Intraclass Correlation Coefficient (ICC), each capturing different aspects of rater agreement:

ICC(C,k): This metric evaluates the consistency of ratings when averaged across all assessors. It is appropriate when the focus is on the reliability of the mean rating, assuming that systematic differences between assessors (e.g., one assessor consistently rating higher or lower) are not of primary concern.

ICC(2,1): This metric assesses the absolute agreement between individual assessors, considering both random and systematic differences. It is suitable when each assessor's rating is of interest, and when assessors are considered representative of a larger population.

By utilizing both ICC(C,k) and ICC(2,1), we provide a comprehensive assessment of inter-rater reliability, capturing both the consistency of average ratings and the agreement among individual assessors.

### Secondary analysis

In our secondary analysis, we compared the time from needle skin puncture to successful needle-target site saline injection between the two needle insertion techniques. Given that some procedures were censored due to incomplete injections, we employed a Cox proportional hazards model with robust sandwich estimates for the covariance matrix to account for potential intra-subject correlation and model misspecification. This approach allowed us to estimate hazard ratios with corresponding 95% Confidence Intervals, providing a measure of the relative efficiency of each technique in achieving the target site. It is important to note that the procedural time did not include the setup of the Infiniti Plus (In-plane, CIVCO Medical Solutions, Coralville, Iowa), which may have influenced overall efficiency, particularly in time-sensitive clinical settings.

Given the limited number of pre-specified secondary outcomes and the exploratory nature of these analyses, we did not apply formal corrections for multiple comparisons. We recognize the potential for increased Type I error and have interpreted these findings with appropriate caution.

### Exploratory analysis

The number of attempts to complete a paravertebral block was summarized by means ± SDs and counts (%). Procedural difficulty was summarized as means ± SDs. The adjudicators’ overall perceptions of needle visibility were also summarized and reported. The inter-rater reliability of overall perception consistency was reported using the same method of calculating inter-rater reliability of percent perfect visualization. All needle insertions were performed under dynamic ultrasound guidance, with real-time visualization throughout needle advancement.

### Sample size

Our study was designed to have 80% power at the 0.05 significance level to detect an absolute 20% difference in perfect needle visibility between needle insertion technique groups. In a previous study, perfect needle visibility with ultrasound was achieved 55% (SD 26%) without mechanical guidance.^17^ Assuming that each resident performed 20 paravertebral blocks and a moderate intra-class correlation coefficient of 0.3, at least 20 residents needed to perform 20 blocks each to detect a 20% absolute difference in needle visibility.

Given the observed SD of 17% and ICC of 0.25, the study had 76% power to detect a 20% absolute difference with the existing sample size. This marginal underpowering may diminish sensitivity to small effect sizes and increase Type II error risk.

Primary and secondary hypotheses were evaluated under a significance criterion of 0.05. All analyses were completed using SAS version 9.4 (SAS Institute, Cary, NC, USA) and *R* statistical software version 3.3.2 (The *R* Foundation for Statistical Computing, Vienna, Austria). The inter-rater reliability test was conducted using the “icc” function in “irr” package in *R*.

### Power analysis

Prior to the study, we conducted an a priori power analysis to determine the necessary sample size to detect a clinically meaningful difference in perfect needle visibility between the needle guidance methods. Assuming a baseline visibility rate of 55% (SD 26%) without mechanical guidance, an anticipated absolute improvement of 20%, an intra-class correlation coefficient of 0.3, and a significance level of 0.05, we calculated that enrolling at least 20 residents, each performing 20 blocks, would provide 80% power to detect the specified difference.

After data collection, we performed a post hoc power analysis to assess the actual power achieved based on observed data. With 19 residents completing the study, an observed standard deviation of 17%, and an intra-class correlation coefficient of 0.25, the post hoc analysis indicated a power of 76% to detect a 20% absolute difference in needle visibility. While slightly below the initial target, this level of power is considered acceptable for exploratory research.

It's important to note that while a priori power analysis is essential for study planning and is widely endorsed, post hoc power analysis is more controversial and should be interpreted with caution.

## Results

We recruited 21 residents, but two were excluded because of a video recording failure. Therefore, 19 residents’ blocks on 17 cadavers were analyzed. The number of cadavers was less than the number of residents since two cadavers were used by two residents each at separate times.

A total of 19 residents successfully completed the study, with 10 (53%) being CA-1 and 9 being CA-2. The cadavers had a mean ± SD BMI of 24 ± 4 kg.m^-2^, with 9 (53%) males and 8 females. Assessments were available for 186 blocks completed with combined mechanical and ultrasound needle guidance, and 188 completed with only ultrasound guidance.

### Primary analysis results

**Figure 3:**
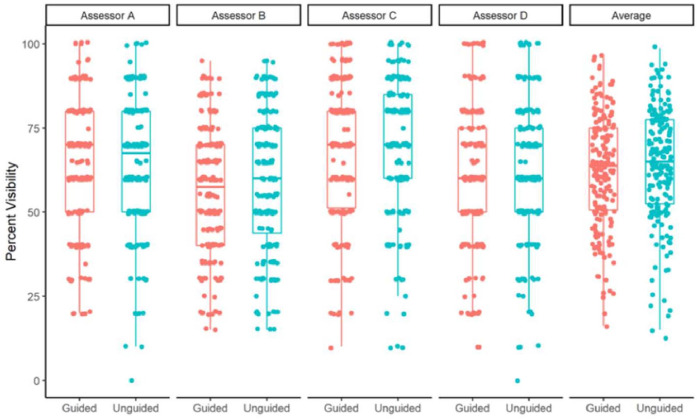
Percent variability for blocks with and without mechanical needle guidance. Assessor A, B, C, D represents four assessors.

The mean percentage of perfect visualization was 62% (SD 17%) for blocks performed with mechanical guidance and 64% (SD 18%) for blocks performed with ultrasound alone (Fig. 3). There was no statistically significant difference in the percent perfect visualization between the two techniques, with the difference (mechanical guidance vs. without) estimated as -1.8% (95% CI [-4.2%, 0.6%], p = 0.15).

Overall consistency on perfect visualization among the 4 assessors was good at 0.85 (95% CI: 0.82, 0.87). This means using the average of 4 assessors’ results as the final percentage perfect visualization was reliable.

The sensitivity analysis using each evaluator’s assessments of percent perfect visualization separately for the comparison between guided method and unguided method showed a similar result to our primary analysis for three evaluators, that no statistically significant difference was detected between the two methods (Table 1). Except for one evaluator, we found the guided method had a lower percentage of perfect visualization with an estimated mean difference of -3.7% (95% CI: [-6.6, -0.7], p = 0.01) comparing guided to unguided insertion.

**Table 1:** Summary of results (subject n = 19).

Outcomes	Needle Guided^d^ (n = 190)	Unguided^d^ (n = 190)	Missing	Difference (95% CI)	p-value
Primary Analysis^a^					
Percent perfect visualization, averaged	62 ± 17	64 ± 17	10	-1.8 [-4.2, 0.6]^a^	0.15
Sensitivity Analysis					
Percent perfect visualization, separately					
Assessor A	64 ± 19	64 ± 19	6	0.6 [-1.9, 3.0]^a^	0.65
Assessor B	56 ± 19	59 ± 21	6	-3.7 [-6.6, -0.7]^a^	0.01
Assessor C	68 ± 21	70 ± 20	6	-1.7 [-4.8, 1.4]^a^	0.29
Assessor D	61 ± 21	63 ± 20	10	-2.4 [-6.1, 1.3]^a^	0.21
Secondary Analysis^b^					
				**Hazard ratio**	
Time to target (seconds)	34 [30, 39]	35 [31, 40]	2	1.00 [0.86, 1.16]	0.99
Exploratory Analysis					
Overall perception of needle visualization, averaged	3 ± 1	3 ± 1	11		
Sensitivity Analysis					
Overall perception of needle visualization, separately					
Assessor A	3 ± 1	3 ± 1	6		
Assessor B	3 ± 1	3 ± 1	6		
Assessor C	3 ± 1	3 ± 1	6		
Assessor D	3 ± 1	3 ± 1	11		
Number of Attempts	1.2 ± 0.6	1.3 ± 1.1	2		
1	162 (85)	160 (84)			
2	18 (9)	19 (10)			
> 3	10 (5)	11 (6)			
Procedural difficulty^c^	6.5 ± 1.9	6.6 ± 1.5			

a Difference is assessed as mean difference through the generalized linear model using GEE (Generalized Estimation Equation) method assuming exchangeable correlation within subjects.

b Guide system effect was assessed as hazard ratio through Cox-proportional survival model using sandwich estimator for covariance matrix to account for within-subject correlation. The summary statistics of each group was median time to target with 95% CI.

c Procedural difficulty of needle-guided systems/without needle-guided systems were rated at subject level, which ranged from 1 to 10, with 1 = easy and 10 = extremely difficult.

d Summary statistics were calculated by simply treating each target as independent and represented as mean ± SD, median [Q1, Q3], or n (%). The total N at each column represent the number of blocks.

### Secondary analysis results

**Figure 4:**
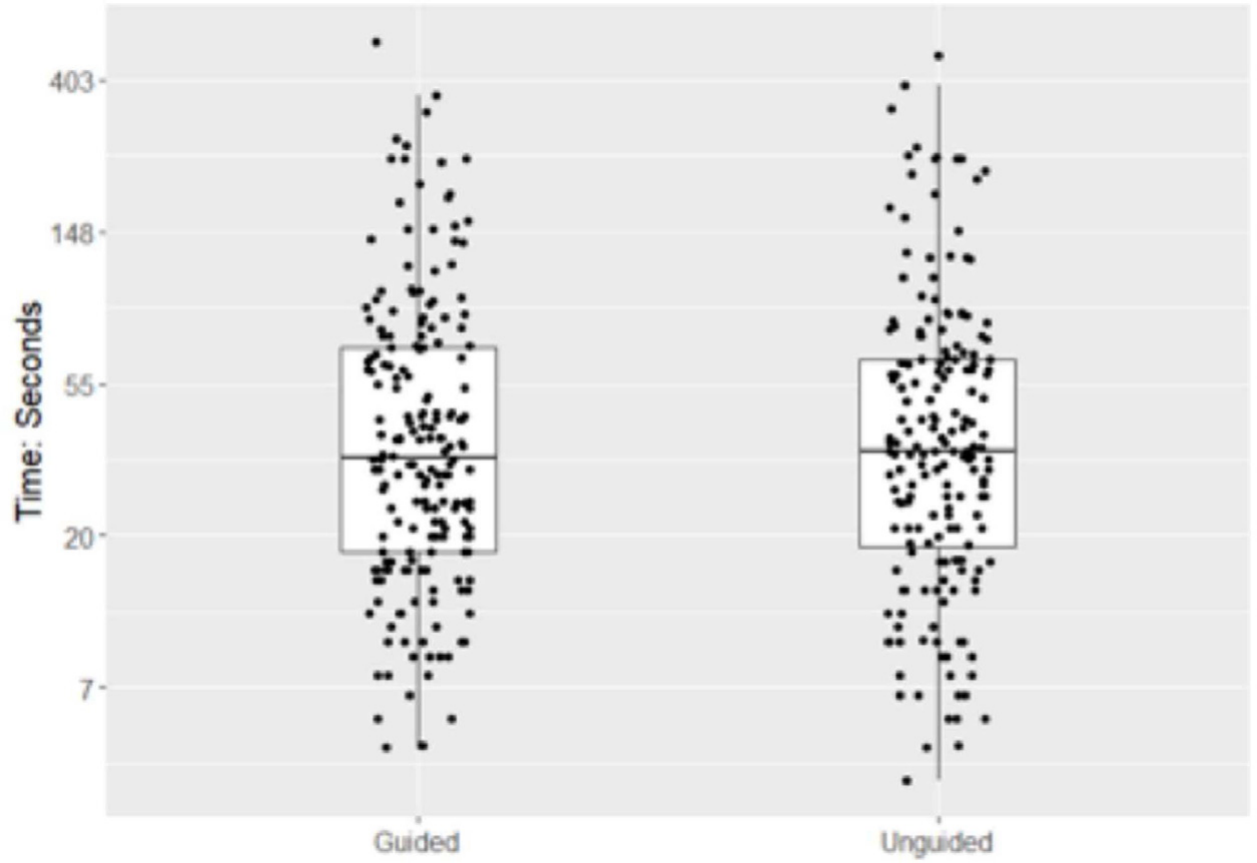
Time to reach target in blocks using needle guidance system vs. without needle guidance.

The median time to finish the block was 34 (IQR: 18, 69) seconds for blocks performed under the needle guidance system and 35 (19, 65) seconds for blocks performed without the needle guidance (Fig. 4). Time to reach the target also did not differ significantly with an estimated hazard ratio of 1.00 (95% CI: [0.86, 1.16], p = 0.99). Finally, time to reach the target site did not differ significantly between groups (95% CI: 14% slower to 16% faster), indicating no clear difference compared to the unguided group.

### Exploratory analysis results

Averaged assessors’ overall perception was estimated as 3 ± 1 in both groups (Table 1). Estimated inter-rater reliability of consistency of 0.84 (95% CI: 0.81, 0.86) on overall perception rating, indicates good consistency among assessors. The evaluator’s overall perception was also summarized separately by treatment groups in Table 1. The residents' procedural difficulty was rated 6.5 ± 1.9 under needle guidance and 6.6 ± 1.5 without needle guidance. The average number of attempts needed to perform each block was 1.2 ± 0.6 with mechanical guidance, and 1.3 ± 1.1 without (Table 1).

Data from all assessors were analyzed; missing values were excluded from the analysis. No imputation methods were applied.

## Discussion

Infiniti Plus (In-plane, CIVCO Medical Solutions, Coralville, Iowa) mechanical needle guidance did not improve ultrasound needle visibility during thoracic paravertebral block by novice residents. Additionally, time to reach the target, and assessors’ overall perception, residents' evaluation, and the average number of attempts were similar with or without the needle guidance.

In a previous study, Infiniti Plus (In-plane, CIVCO Medical Solutions, Coralville, Iowa) needle guidance did not improve the percentage of perfect needle visibility during ultrasound-guided femoral nerve catheter placement.^17^ Additionally, Infiniti Plus (In-plane, CIVCO Medical Solutions, Coralville, Iowa) guidance did not improve the fraction of successful femoral nerve catheter insertions or the number of attempts. However, the median time to properly position femoral nerve catheters was about a minute shorter with guidance, apparently because the device kept the needle tracking towards the target even when ultrasound visibility was imperfect.

Mansour et al.^18^ evaluated a different CIVCO’s needle guide called the Ultra-Pro II. The Ultra-Pro II is a two-part system consisting of custom reusable bracket and a disposable snap-on needle guide. Multi-angle brackets provide different angles appropriate for various blocks. This system improves needle visibility score, and reduces time needed to perform a parasagittal in-plane thoracic paravertebral block. The number of needle passes was lower with guidance than without. Physician and patient satisfaction were better when using the needle guide.

The Infiniti Plus (In-plane, CIVCO Medical Solutions, Coralville, Iowa) needle guide that we used in our study has a fixed angle which sometimes made needle manipulation difficult. The fixed angle may explain why the Infiniti Plus (In-plane, CIVCO Medical Solutions, Coralville, Iowa) guide did not improve the evaluator’s overall perception of needle visibility, time to reach the target site, the number of attempts, and procedural difficulty evaluated by novice residents. Additionally, previous trials of the Infiniti Plus and Ultra-Pro^17^^,^^18^ involved experienced anesthesiologists, whereas our procedures were conducted by novice residents.

Gupta et al.^16^ reported also that Ultra-Pro II multi-angle in-plane needle guidance reduces median time to complete a simulated nerve targeting task in a phantom gel simulation of the regional block by novice sonographers by 27%, and the needle guide system provided improved needle view at the completion of the task by a factor of three. Both that study and ours used novice sonographers, but their phantom gel simulation may not entirely reflect needle insertion in human tissue planes.

The Infiniti Plus system is shown to offer an open-channel design with infinite angle adjustability and broad-gauge compatibility via a snap-on disposable guide and reusable bracket, optimized for in-plane procedures such as biopsies, fluid aspiration, and catheter placement. By contrast, the Ultra-Pro II employs a multi-angle bracket with discrete insertion angles and a larger quick-release tab, accepting a slightly different gauge range and offering alternative cover formats tailored for regional blocks and line placements.

The comparison clarifies that Infiniti Plus prioritizes continuous angle flexibility and broad-gauge acceptance, whereas Ultra-Pro II emphasizes preset entry angles and an enhanced quick-release feature, each addressing different clinical workflow preferences.

While mechanical guides simplify in-plane alignment, fixed-angle systems can impede ergonomic probe handling and real-time needle redirection, particularly in obliquely angled or deep thoracic paravertebral blocks where minor trajectory adjustments are crucial.^19^^,^^20^ The rigid sleeve may force suboptimal wrist postures and limit probe tilt, increasing musculoskeletal strain and reducing beam-needle alignment efficiency.^21^ In parasagittal approaches to the paravertebral space, where navigation around ribs and variable tissue depths demands frequent small angulation changes, fixed guides may hinder nuanced needle advancement, potentially prolonging procedure time or risking misplacement.^22^. We suggest that variable-angle or multi-angle guides could offer superior adaptability and ergonomics for thoracic blocks.

Several complementary technologies beyond the Infiniti Plus mechanical guide have been developed to enhance ultrasound needle visualization: echogenic needles, which feature surface coatings or textured grooves to increase backscatter and markedly improve needle tip conspicuity in vivo, especially at steep insertion angles,^23^^,^^24^ electronic beam steering, wherein the ultrasound beam is dynamically tilted to maintain near-perpendicular incidence on the needle shaft, has been shown to significantly enhance needle and tip visibility by subjective inspection;^25^ active needle-tracking systems like Onvision employ a piezoelectric sensor near the needle tip and provide real-time visual overlays (e.g., colored circles) on the ultrasound screen, improving tip localization in infraclavicular and other regional blocks.^26^

Mechanical needle guides may be a valuable bridging tools for novices by providing consistent entry angles and reducing cognitive load during the earliest stages of ultrasound-guided block training. However, reliance on structured guides risks delaying the acquisition of proprioceptive skills and intuitive probe-needle coordination that are essential for independent free-hand practice. To ensure balanced skill development, we recommend integrating mechanical guidance into a graduated curriculum, with early use under expert supervision, followed by systematic weaning toward unguided techniques, to reinforce both anatomical understanding and hand-eye alignment proficiency.

While our cadaver study showed no benefit of the Infiniti Plus mechanical guide in novice resident-performed thoracic paravertebral blocks, mechanical guidance has been shown to improve first-pass success and reduce needle passes in peripheral nerve blocks such as femoral and sciatic blocks, enhancing procedural efficiency and safety.^27^^,^^28^ It can also stabilize needle trajectory during central venous catheterization, reducing inadvertent vessel punctures and improving placement accuracy in both emergency and routine vascular access.^29^^,^^30^ Mechanical guides further aid deep or technically demanding blocks, like lumbar plexus and quadratus lumborum, by maintaining consistent in-plane alignment at steep insertion angles, thereby enhancing needle tip visibility.^31^^,^^32^ Experienced anesthesiologists may benefit from reduced ergonomic fatigue and improved precision during prolonged procedures or challenging anatomies, as mechanical guidance mitigates inadvertent beam-angle deviations caused by operator fatigue or transducer movement.^33^^,^^34^

A limitation of our study was that some information regarding the cadavers was incomplete. For example, 5 were missing BMI and the sex of two was unknown. A limitation of any study in cadavers is the physiological differences between living and deceased tissues. We mitigated this shortcoming by using unembalmed human cadavers at room temperature to simulate the tissue elasticity of live humans. Cadaver studies allowed many procedures to be tested by novice residents which is not possible in patients. Evidence from cadavers may not directly predict clinical outcomes, thus cautious interpretation of the results in cadavers is warranted, and future study in patients in a randomized trial is necessary. Additionally, we did not record needle guidance system set-up time, although set-up time contributes to overall procedure duration. Our aim was to evaluate the usability of mechanical needle guidance among novice residents; participants were intentionally selected as inexperienced in ultrasound-guided techniques. We assume that procedural skills improved over the course of the study, potentially influencing needle visualization outcomes. Anatomical differences across the various cadavers and spinal levels were considered to ensure that our results reflect real-world applicability rather than being limited to findings in cadavers.

The lack of significant difference between guided and unguided methods may reflect ceiling effects, given that both approaches were already relatively successful in reaching the target site.

Although cadaver models offer high‐fidelity anatomy and safe practice conditions, they lack several critical in vivo characteristics, namely real‐time bleeding, respiratory excursion, and native tissue turgor, which can substantially affect needle handling and visualization during regional anesthesia.^35^^,^^36^ The absence of bleeding removes the challenge of managing artifact and hemodynamic changes seen during live procedures, while static lungs preclude practicing needle redirection under respiratory motion. Moreover, postmortem tissue dehydration and fluid shifts alter normal turgor, potentially overestimating needle tip conspicuity and underrepresenting the force required for ligament penetration. Consequently, our negative findings in a cadaver setting may not fully translate to clinical practice, and future work should validate mechanical guidance systems under live conditions or with dynamic cadaver preparations (e.g., perfused, ventilated, or Thiel‐embalmed models) to better simulate real‐world challenges.^37^

Our study’s achieved power of 0.76, just below the 0.80 convention, reflects a slight underpowering due to exclusion of two residents. Borderline underpowered trials are prone to Type II errors, whereby true effects may go undetected, and thus our non-significant results should be interpreted with appropriate caution. We recommend that subsequent investigations aim for larger sample sizes or multicenter designs to ensure adequate power for detecting clinically relevant differences.

We did not evaluate within-participant performance over the sequential blocks, so it remains possible that residents improved with practice in a way that could mask or modify any early advantage of the mechanical guide. Future studies should incorporate formal learning-curve analyses, such as comparing early versus late block performance, to clarify whether guidance systems confer the greatest benefit during initial skill acquisition.

Some outcomes in our study, such as procedural difficulty and needle visualization scores, relied on subjective assessment. While blinding of assessors was implemented to reduce bias, subjective scoring inherently introduces variability and potential bias. This limitation should be considered when interpreting our findings.

## Conclusion

In this cadaveric model of thoracic paravertebral blocks performed by novice residents, the Infiniti Plus mechanical guide did not confer measurable improvements in needle visualization, procedural efficiency, or ease of block insertion compared to freehand technique. These findings contrast with prior evidence suggesting that certain mechanical guides, particularly variable- or multi-angle systems, may enhance first-pass success and reduce procedure time in other block settings. The fixed-angle design of the Infiniti Plus may limit ergonomic flexibility and hinder nuanced adjustments required for complex thoracic approaches.

Although our results did not demonstrate clear benefits, mechanical guidance devices may still serve a role in early training by providing consistent entry angles and reducing cognitive load for beginners. However, their utility should be balanced against the risk of slowing the development of freehand skills critical for independent practice. Future research should validate these findings in live patient models, with larger multicenter samples and formal learning-curve analyses, to determine whether mechanical guidance systems hold value in clinical training or specific block types. Ultimately, careful integration of such tools into structured educational curricula may help optimize both novice learning and procedural safety.

## Data availability statement

The datasets generated and/or analyzed during the current study are available from the corresponding author upon reasonable request.

## Authors’ contributions

All listed authors have made significant contributions to this work, including study design, data collection, analysis, interpretation, and manuscript preparation. Their collective efforts justify their recognition as authors.

## Declaration of competing interest

The authors declare no conflicts of interest.

## Appendix Supplementary materials

Image, video 1

Image, application 2

Image, application 3
