# Supplementary material for: Mechanical needle guidance for ultrasound-guided parasagittal oblique in-plane paravertebral blocks: a cadaveric study
Source: Braz J Anesthesiol. 2025 Nov 28;76(1):844716. doi: 10.1016/j.bjane.2025.844716 (PMC12811454; doi:10.1016/j.bjane.2025.844716)

**BJAN-D-25-00089_Supplementary Material 1**

**Supplemental Material 1** Examination on the anatomy and ultrasound imaging paravertebral block.

**Paravertebral Block**

**Questions:**

1. All of the following statements are true regarding the boundaries of paravertebral space except

A. The boundaries of the three-sided wedge—posterior, medial boundary, and anterolateral— extend caudally and Cephalad, as the segmental spaces communicate up and down.

B.The PVS is bounded posteriorly by transverse processes, the rib heads.

C. The medial boundary is the vertebral body, the intervertebral disks, and the intervertebral foramen at each level.

D. The anterolateral boundary is the parietal pleura.

E. Laterally, the space tapers and closed.

2. All of the following are indications of thoracic Paravertebral block except?

A. Thoracic surgery

B. Breast surgery

C. Cholecystectomy, upper abdominal surgeries

D. Knee surgery

E. Renal and ureteric surgery

3. All of the following are advantage of (PV) block compared to epidural block except?

A. PVB associated with less urinary retention

B. PVB associated with less PONV

C. PVB associated with less hypotension

D. PVB had less pulmonary complications

E. All of the Above

4-. The following statement is true or false: The spinal nerves in this space are devoid of a fascial sheath, making them susceptible to local anesthetics.

1-True

2-False

5- Which statement about the Local Anesthetic Spread in the PV space is MOST likely true?

1. PVS communicates with spaces above and below
2. 15 – 20 ml injections cover approximately 4 dermatomes
3. Accumulation of bupivacaine occur during continuous paravertebral infusion without clinical signs of toxicity
4. The addition of 5 mcg/ml epinephrine to ropivacaine significantly delays its systemic absorption
5. The absorption of ropivacaine after TPVB is described by rapid and slow absorption phases.
6. All of the above

6- In Classic PVB Landmark Approach Which of the following is MOST likely true?

A. Needle insertion 2.5 to 3 cm lateral to the cephalad edge of spinous process.

B. The needle advanced perpendicular to the skin until transverse process contacted

C. Needle advanced 1-1.5 cm

D. Pop or click may be felt just prior to entry into PVS

E. All of the above

7-In this ultrasound image from the thoracic paravertebral region please identify the following:

1. Thoracic paravertebral space
2. Pleura
3. Paraspinal muscles
4. Superior costo-transverse ligament
5. Lung


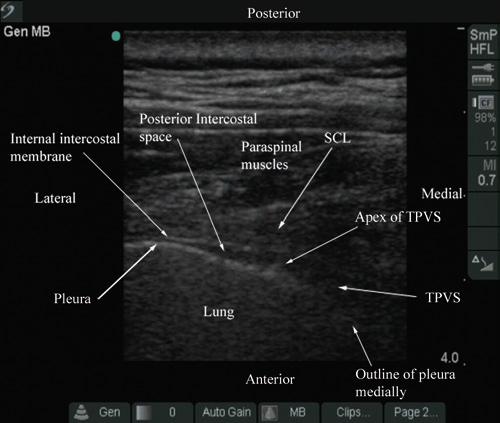


8-In this ultrasound image from Real- time USG TPVB , Which approach used in the image:

1. Transverse Scan with Short-Axis Needle Insertion
2. Paramedian Oblique Sagittal Scan with In-Plane Needle Insertion
3. Transverse Scan with In-Plane Needle Insertion
4. The Intercostal Approach to the TPVS
5. None of the above


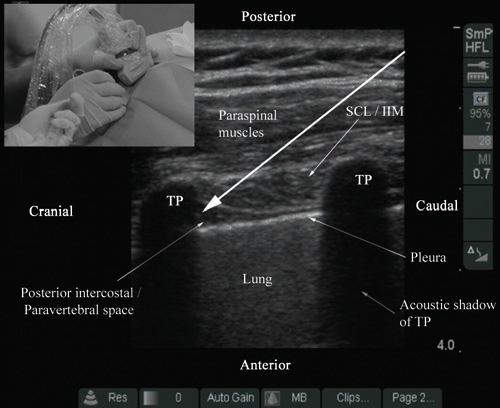


9-Which of the following is the most important ultrasonographic sign for successful PVB?

A. Widening of the paravertebral space

B. Anterior displacement of the pleura

C. Spread of local anesthetic (LA) to the posterior intercostal space

D. Increased echogenicity of the pleura

E. All of the Above

10-In this image from the thoracic paravertebral region please identify the following:

A. Thoracic paravertebral space

B. External intercostal muscle

C. Internal intercostal membrane

D. Endothoracic fascia

E. Ventral ramus

F. Sympathetic chain


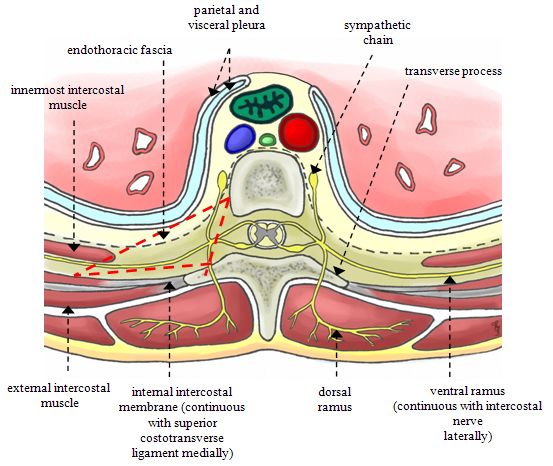

Supplement: Supplementary file 2 [file mmc2.docx]
