# Supplementary material for: Mechanical needle guidance for ultrasound-guided parasagittal oblique in-plane paravertebral blocks: a cadaveric study
Source: Braz J Anesthesiol. 2025 Nov 28;76(1):844716. doi: 10.1016/j.bjane.2025.844716 (PMC12811454; doi:10.1016/j.bjane.2025.844716)
Supplement: Supplementary file 3 [file mmc3.docx]

**BJAN-D-25-00089_Supplementary Material 2**

**Supplemental Material 2** Video of ultrasound-guided paravertebral block using paramedian oblique sagittal scan with needle guidance at T10.
